# Sensitization by Pulmonary Reactive Oxygen Species of Rat Vagal Lung C-Fibers: The Roles of the TRPV1, TRPA1, and P2X Receptors

**DOI:** 10.1371/journal.pone.0091763

**Published:** 2014-04-03

**Authors:** Ting Ruan, Yu-Jung Lin, Tien-Huan Hsu, Shing-Hwa Lu, Guey-Mei Jow, Yu Ru Kou

**Affiliations:** 1 School of Medicine, Fu Jen Catholic University, New Taipei City, Taiwan; 2 Department of Physiology, School of Medicine, National Yang-Ming University, Taipei, Taiwan; 3 Department of Veterinary Medicine, National Chung-Hsing University, Taichung, Taiwan; 4 Department of Urology, Taipei City Hospital, Zhong-Xiao Branch, Taipei, Taiwan; 5 Department of Physiology, School of Medicine, Chung Shan Medical University, Taichung, Taiwan; Indiana University School of Medicine, United States of America

## Abstract

Sensitization of vagal lung C-fibers (VLCFs) induced by mediators contributes to the pathogenesis of airway hypersensitivity, which is characterized by exaggerated sensory and reflex responses to stimulants. Reactive oxygen species (ROS) are mediators produced during airway inflammation. However, the role of ROS in VLCF-mediated airway hypersensitivity has remained elusive. Here, we report that inhalation of aerosolized 0.05% H_2_O_2_ for 90 s potentiated apneic responses to intravenous capsaicin (a TRPV1 receptor agonist), α,β-methylene-ATP (a P2X receptor agonist), and phenylbiguanide (a 5-HT_3_ receptor agonist) in anesthetized rats. The apneic responses to these three stimulants were abolished by vagatomy or by perivagal capsaicin treatment, a procedure that blocks the neural conduction of VLCFs. The potentiating effect of H_2_O_2_ on the apneic responses to these VLCF stimulants was prevented by catalase (an enzyme that degrades H_2_O_2_) and by dimethylthiourea (a hydroxyl radical scavenger). The potentiating effect of H_2_O_2_ on the apneic responses to capsaicin was attenuated by HC-030031 (a TRPA1 receptor antagonist) and by *iso*-pyridoxalphosphate-6-azophenyl-2′,5′-disulphonate (a P2X receptor antagonist). The potentiating effect of H_2_O_2_ on the apneic responses to α,β-methylene-ATP was reduced by capsazepine (a TRPV1 receptor antagonist), and by HC-030031. The potentiating effect of H_2_O_2_ on the apneic responses to phenylbiguanide was totally abolished when all three antagonists were combined. Consistently, our electrophysiological studies revealed that airway delivery of aerosolized 0.05% H_2_O_2_ for 90 s potentiated the VLCF responses to intravenous capsaicin, α,β-methylene-ATP, and phenylbiguanide. The potentiating effect of H_2_O_2_ on the VLCF responses to phenylbiguanide was totally prevented when all antagonists were combined. Inhalation of 0.05% H_2_O_2_ indeed increased the level of ROS in the lungs. These results suggest that 1) increased lung ROS sensitizes VLCFs, which leads to exaggerated reflex responses in rats and 2) the TRPV1, TRPA1, and P2X receptors are all involved in the development of this airway hypersensitivity.

## Introduction

Patients with asthma display airway hypersensitivity, which is characterized by exaggerated sensory and reflex responses to inhaled irritants and chemical mediators [1]–[3]. Asthmatic lungs also exhibit increased oxidative stress, which is partly due to excess production of reactive oxygen species (ROS) [4], [5]. The majority of the ROS are superoxide anion radicals, hydrogen peroxide (H_2_O_2_) and hydroxyl radicals (•OH) [6]. The superoxide anion radical dismutates to form H_2_O_2_, which in the presence of iron can further react to form more reactive •OH via the Fenton reaction [6]. Indeed, the level of H_2_O_2_, a relatively stable ROS, in exhaled breath condensate has been shown to be increased in patients with asthma [7], [8]. While ROS have been suggested to play a role in the pathogenesis of asthma [4], [5], their involvement in the development of the airway hypersensitivity remains elusive.

Vagal lung C-fibers (VLCFs) are stimulated by a variety of chemical mediators or inhaled irritants; this, in turn, causes airway irritation and triggers an array of defense reflex responses such as apnea, coughing and bronchoconstriction [9], [10]. Thus, sensitization of VLCFs by inflammatory mediators has been implicated in the pathogenesis of the airway hypersensitivity in asthma patients [2], [11]–[13]. To this end, three previous studies have provided indirect evidence indicating that there is a cause-effect relationship between ROS and the airway hypersensitivity. Shen et al. [14] reported that acute intermittent hypoxia augmented the afferent and reflex responses to stimulants of VLCFs in rats. Tsai et al. [15], [16] demonstrated that laryngeal acid-pepsin insult augmented the reflex responses to stimulants of the superior laryngeal C-fibers in rats. The exaggerated reflex responses observed in these studies can be prevented by pretreatment with antioxidants, indicating the possible involvement of ROS in the sensitization of airway C-fibers and the development of airway hypersensitivity. However, direct evidence suggesting that an increase in lung level of ROS may sensitize VLCFs leading to exaggerated reflex responses remains lacking.

Investigations targeting the sensory responses of VLCFs to agonists have revealed that their nerve terminals may express a range of pharmacological receptors [9], [10]; these include the transient receptor potential vanilloid 1 (TRPV1), the transient receptor potential ankyrin 1 (TRPA1), and the P2X receptors [17]–[21]. In this context, a direct increase in the lung level of ROS by airway challenge with aerosolized H_2_O_2_ has been shown to stimulate VLCFs via activation of the TRPV1, TRPA1, and P2X receptors [20]–[23]. Additionally, studies investigating the responses of VLCFs to oxidants have suggested that ROS derived from the oxidants may activate these three types of receptors [18], [24]–[27]. Importantly, it has been proposed that these three types of pharmacological receptors have roles in the development of the airway hypersensitivity that is mediated by airway C-fibers [1], [2], [14], [15], [28], [29]. Taken together, these lines of evidence lend support to the possibility that the TRPV1, TRPA1, and P2X receptors are likely to play contributory roles in ROS-induced VLCF-mediated airway hypersensitivity. Nevertheless, this possibility remains to be explored.

The present study was undertaken to investigate the following: 1) whether airway exposure to aerosolized H_2_O_2_ may exaggerate the reflex and afferent responses of VLCFs to intravenous injections of chemical stimulants in anesthetized rats and 2) whether the TRPV1, TRPA1, and P2X receptors play contributory roles in this ROS-mediated airway hypersensitivity. To achieve these goals, the study of reflex apneic responses was performed by measuring the respiratory parameters of spontaneously breathing rats. In parallel, a study of the afferent responses was conducted by measuring the activity of single unit VLCF of ventilator-assisted rats.

## Materials and Methods

The procedures described below were carried out in accordance with the recommendations found in the *Guide for the Care and Use of Laboratory Animals* published by the National Institute of Health, USA and were approved by the Institutional Animal Care and Use Committee of Chung Shan Medical University, Taichung, Taiwan (Permit Number: 2008-702).

### Animal preparation

Male Sprague-Dawley rats were anesthetized with an intraperitoneal injection of chloralose (100 mg/kg; Sigma Chemical, St Louis, MO) and urethane (500 mg/kg; Sigma) dissolved in a borax solution (2%; Sigma). During the course of the experiments, supplemental doses of chloralose (20 mg/kg/h) and urethane (100 mg/kg/h) were administered to maintain the abolition of the corneal reflex as well as the abolition of the pain reflex induced by pinching the animal's tail. The rats were tethered in a supine position, and a short tracheal cannula (PE-260) was inserted just below the larynx via a tracheostomy. A PE-50 catheter was inserted into the jugular vein and advanced until the tip was close to the right atrium to allow intravenous administration of pharmacological agents. One femoral artery was cannulated in order to record arterial blood pressure and heart rate. The animal's neck was opened along the midline and both cervical vagus nerves were carefully isolated from their connective tissues. For the reflex studies, the animals were allowed to breath spontaneously through the tracheal cannula. Respiratory flow (VR) was measured with the pneumotachograph (Fleisch, 4/0, Richmond, VA) that was coupled to a differential pressure transducer (Validyne MP45-12, Northridge, CA). The flow signal was integrated to give tidal volume (VT). For the electrophysiological studies, rats received a midline thoracotomy, and both vagus nerves were ligated just above the diaphragm in order to eliminate afferent signals arising from the lower visceral organs. The lungs were then ventilated with a rodent respirator (Harvard 683, South Natick, MA) at a constant volume of 8 ml/kg with a positive end-expiratory pressure of 3 cmH_2_O. The frequency of the respirator was set at 50 breaths/min and was kept constant in each experiment. The tracheal pressure was monitored using a pressure transducer (Validyne MP45-28) via a side tap to the tracheal cannula. All physiological signals were recorded on a chart recorder (Gould TA11, Cleveland, OH) and a tape recorder (Neurocorder DR-890, New York, NY) for later analysis. Body temperature was maintained at about 37°C throughout the experiment by means of a servo-controlled heating blanket.

### Experimental treatment of vagus nerves

The technique of perivagal capsaicin treatment was similar to that reported previously and was employed to selectively block the conduction of the VLCFs [30], [31]. In brief, a segment (∼3 mm) of each cervical vagus nerve was wrapped in a cotton strip presoaked in a capsaicin solution (0.25 mg/ml). After 20 min, when the reflex responses to an intravenous injection of capsaicin (1 µg/kg) had been abolished, the cotton strips were removed. The blocking effect of the perivagal capsaicin treatment on the reflex responses to capsaicin injection has been shown to last for 80–120 min [31]. To perform perivagal sham treatment, a segment of each cervical vagus nerve was wrapped for 20 min in a cotton strip which had been presoaked in the vehicle of the capsaicin solution. To perform vagotomy, both vagus nerves were sectioned at the cervical level.

### Recording of C-fiber activity

Neural activity arising from VLCFs were recorded using techniques that have been described elsewhere [21], [32]. Briefly, a fine afferent filament of the right vagus nerve was split and placed on a unipolar recording electrode to record afferent nerve activity. The thin filament was further split until the afferent activity arising from a single unit was electrically isolated. Once the presence of a suspected single unit was detected, capsaicin (1 µg/kg) was injected as a bolus into the right atrium. Only afferent fibers that showed stimulation within 2 s after the injection were studied. The conduction velocities of the VLCFs identified by this method have been shown to be within the range of C fibers [33], [34]. The general locations of all fibers were identified by their responses to the gentle pressing of the lungs with a saline-wetted cotton Q-tip.

### Sensitization of the airways through the use of aerosolized H_2_O_2_

The generation and delivery of aerosolized H_2_O_2_ were achieved by methods that have been described previously [21], [23]. Briefly, two concentrations (0.025% and 0.05%) of a H_2_O_2_ solution were prepared just prior to each set of experiments by mixing 35% H_2_O_2_ (Shimakyu, Osaka, Japan) with phosphate buffer solution (PBS) to the desired concentration with the pH adjusted to 7.4. H_2_O_2_ or PBS aerosol was then generated by a micropump nebulizer (Aeroneb® Lab Nebulizer System, Aerogen, Galway, Ireland) that contained either H_2_O_2_ or PBS solution. In the reflex studies, the H_2_O_2_ or PBS aerosol was delivered at a constant flow rate of 3 ml/s and the aerosol was spontaneously inhaled into the lower airways and lungs for a 90-s period through a side arm of the tube (8 mm ID) connected to the outlet of the nebulizer [23]. In the electrophysiological studies, the H_2_O_2_ or PBS aerosol was delivered by the respirator for a 90-s period using a circuit described previously [21].

### Preparation of bronchoalveolar lavage fluid (BALF)

The BALF samples from rats were pooled on the day of ROS measurement. Each anesthetized rat was surgically inserted with a tracheal cannula. At a time point 1 min after 90 s H_2_O_2_ (0.05% H_2_O_2_) or PBS challenge, PBS was intratracheally instilled into the lungs and then the lungs were lavaged three times using 3 ml PBS. The level of ROS in the BALF was measured immediately after collection of BALF samples.

### Measurement of levels of ROS in the BALF

The ROS level in the BALF supernatants was measured by luminol-enhanced chemiluminescence (CL) using a method described previously [35]. The changes in CL intensity (mV) were continuously monitored (Bio Orbit 1251; Bio Orbit, Turku, Finland). The CL of the BALF (0.4 ml) was recorded for 60 s to give a baseline reading and then luminol (5-amino-2,3-dihydro-1,4-phthalazinedione) was added to a final concentration of 2.4×10^−5^ M in order to enhance oxygen radical production, which was then recorded for 240 s. The CL counts are given in units/s.

### Pharmacological agents

Solutions of the various pharmacological agents at the desired concentrations were prepared daily. For stimulation of the VLCFs, capsaicin (0.5 µg/kg; a TRPV1 receptor agonist) [21], α,β-methylene-ATP (α,β-meATP; 10 µg/kg; a P2X receptor agonist) [22], and phenylbiguanide (5 µg/kg; a 5-HT_3_ receptor agonist) [17] with a volume of 0.1 ml were injected as a bolus into the right atrium; these drugs were first injected into the catheter (dead space = ∼0.2 ml) and then flushed into the circulation by an injection of 0.4 of ml saline. Receptor antagonists for TRPV1 (capsazepine; 3 mg/kg) [21], TRPA1 (HC-030031; 3 mg/kg;) [18], and P2X receptors [*iso*-pyridoxalphosphate-6-azophenyl-2′,5′-disulphonate (*iso*-PPADS); 15 mg/kg] [18], as well as dimethylthiourea (a •OH scavenger; 1 g/kg) [36], with a volume of 0.35 ml, were slowly injected into the jugular vein over a 1-min duration. Catalase, an enzyme that degrades H_2_O_2_ [6] was dissolved in PBS to give a concentration of 13500 IU/ml. An aerosol of catalase or heat-inactivated catalase was generated and inhaled into the lower airways for a period of 5 min using the nebulizer and the circuit for delivery of the H_2_O_2_ aerosol [21]. The doses and treatment times of the drugs used in this study in order to be effective were adopted from previous studies [17], [18], [20], [21], [23] or determined in our preliminary study (Supporting Information Table S1). The vehicle for phenylbiguanide, α,β-meATP, *iso*-PPADS, and dimethylthiourea was saline. The vehicle for capsaicin, capsazepine, and HC-030031 was a solution that contained dimethyl sulfoxide, Tween 80, ethanol and saline. Except for HC-030031 (Tocris Cookson, Ellisville, MO) and *iso*-PPADS (Tocris), all other drugs were purchased from Sigma-Aldrich. The functions, doses, administration routes, and vehicles of these pharmacological agents are summarized in Table S2 (Supporting Information).

### Experimental design and protocols

In this study, 432 rats (weighing 300–380 g) were divided into 54 groups to conduct seven series of experiments; each group consisted of eight rats, and only one VLCF was studied in each rat. An interval of 90 min was allowed to elapse between any two inhalations of H_2_O_2_ or PBS. In study series 1, the reflex apneic responses of LVCFs (*Groups 1–6*) or the afferent responses of LVCFs (*Groups 7–12*) to capsaicin, α,β-meATP and phenylbiguanide individually were studied 1 min after one inhalation of PBS and then 1 min after each of the two inhalations of H_2_O_2_ (0.025% or 0.05%) to give three inhalation for each rat or after each inhalation of PBS, also three inhalation for each rat, in order to assess the sensitizing effect of H_2_O_2_. In study series 2 (*Groups 13–15*), reflex apneic responses to capsaicin, α,β-meATP or phenylbiguanide were studied 1 min after one inhalation of PBS, and then 1 min after and 40 min after H_2_O_2_ sensitization (0.05%); the aim was to examine the reversibility of the H_2_O_2_-induced airway sensitization. In study series 3 (*Groups 16–36*), reflex apneic responses to capsaicin, α,β-meATP or phenylbiguanide were studied 1 min after one PBS inhalation and then 1 min after each of the two H_2_O_2_ sensitization (0.05%). In *Groups 16–24*, three different vagal nerve treatment (perivagal capsaicin treatment, perivagal sham treatment or vagotomy) was carried out 30 min prior to the second H_2_O_2_ sensitization in order to assess the role of the VLCFs in the H_2_O_2_-induced airway sensitization. In *Groups 25–36*, pretreatment with catalase, heat-inactivated catalase, dimethylthiourea or the vehicle of dimethylthiourea were carried out 10 min prior to the second H_2_O_2_ sensitization in order to assess the role of ROS in the H_2_O_2_-induced airway sensitization. In study series 4 (*Groups 37–44*), the reflex apneic responses to capsaicin or α,β-meATP were studied 1 min after one PBS inhalation and then 1 min after each of the two H_2_O_2_ sensitizations (0.05%). Pretreatment with capsazepine, HC-030031, *iso*-PPADS or their vehicles was made at 2, 10, 15, and 15 min, respectively, prior to the second H_2_O_2_ sensitization. In study series 5 (*Groups 45–50*), the reflex apneic responses to phenylbiguanide were studied 1 min after one PBS inhalation and then 1 min after each of the two H_2_O_2_ sensitization (0.05%). Pretreatment with capsazepine, HC-030031, *iso*-PPADS, a combination of capsazepine and *iso*-PPADS, a combination of the three antagonists or their vehicles was made at 2, 10, 15, 15, 15, and 15 min, respectively, prior to the second H_2_O_2_ sensitization. In study series 6 (*Groups 51–52*), the afferent responses of VLCFs to phenylbiguanide were studied 1 min after one inhalation of PBS and then 1 min after each of the two inhalations of H_2_O_2_ (0.05%). Pretreatment with a combination of capsazepine, HC-030031, and *iso*-PPADS or their vehicles was made respectively prior to the second H_2_O_2_ sensitization. Study series 4–6 were conducted in order to assess the role of the TRPV1, TRPA1, and P2X receptors in the H_2_O_2_-induced airway sensitization. In study series 7 (*Groups 53–54*), BALF samples were obtained 60 s after inhalation of PBS or 0.05% H_2_O_2_ to allow later analysis of the ROS levels in the lungs.

### Data analysis and statistics

For the reflex studies, respiratory frequency (f), tidal volume (VT), and expiratory duration (TE) were analyzed on a breath-by-breath basis over at least 20 breaths before and 120 breaths after the injection of stimulants. The baseline TE was calculated as the average value over the 10-breath period immediately preceding a challenge. To compare the apneic response evoked by the various different experimental conditions, the longest TE occurring during the first 10 breaths after a challenge was divided by the baseline TE to yield the apneic ratio. For the electrophysiological studies, C-fiber activity was continuously analyzed at 1-s intervals over at least 20 s before and 120 s after injection of the stimulant. Baseline fiber activity was calculated as the average value over the 10-s period immediately preceding the injection of the stimulant. The peak response was defined as the maximum 5-s average during the 20 s following injection of the stimulant. In all studies, the heart rate and mean arterial blood pressure were continuously analyzed at 1-s intervals. These physiological parameters were analyzed using a computer equipped with an A/D converter (Gould DASA 4600) and software (BioCybernatics, 1.0; Taipei, Taiwan). The results from two groups (baseline values before and after inhalation, and ROS levels) were compared using paired and unpaired *t*-tests. The results from three or more groups were compared using one-way repeated-measure analysis of variance (ANOVA) (data from the studies of the reflex responses) or using two-way repeated-measure ANOVA (data from the studies of the afferent responses), which were followed by Fisher's least significant difference procedure where appropriate. A value of *p*<0.05 was considered significant. All data are presented as the mean ± SE.

## Results

### Baseline physiological parameter findings

In the reflex studies, the mean baseline f and VT values of the 352 rats involved were found to be 72.2±0.6 breaths/min and 1.78±0.02 ml, respectively. In the electrophysiological studies, a total of 64 VLCFs were recorded from 64 rats; these had sparse or no activity during the baseline period with their mean baseline activity being 0.01±0.00 impulses/s. The baseline VT was not significantly affected by inhalation of aerosolized PBS or 0.05% H_2_O_2_ (Table 1). The baseline f and C-fiber activity were also not significantly affected by inhalation of aerosolized PBS, but they were slightly increased after the termination of the inhalation of aerosolized 0.05% H_2_O_2_ (Table 1). At the beginning of experiments, the mean baseline arterial blood pressure and heart rate values of the these 416 rats were 97.3±0.7 mmHg and 364.8±1.8 beats/min, respectively. After completion of the protocol, the mean baseline arterial blood pressure and heart rate values of the same rats remained close to the baseline values at 97.5±0.7 mmHg and 358.5±2.0 beats/min, respectively.

**Table 1:** Baseline respiratory frequency, tidal volume, and fiber activity of vagal lung C-fibers before and immediately after inhalation of aerosolized PBS or 0.05% H_2_O_2_ in anesthetized rats.

		PBS inhalation		0.05% H_2_O_2_ inhalation	
	*n*	Before	After	Before	After
f (breaths/min)	328	72.4±0.6	72.8±0.6	72.0±0.6	75.2±0.6*
VT (ml)	328	1.77±0.02	1.79±0.02	1.83±0.02	1.86±0.02
Fiber activity (impulses/s)	40	0.01±0.01	0.02±0.01	0.01±0.01	0.07±0.02*

Respiratory frequency (f) and tidal volume (VT) were measured using spontaneously breathing rats. Fiber activity was measured using ventilator-assisted rats. Baseline values were averaged over 10 breaths or a 10-s period before and after inhalation. Data in each group are the mean ± SE.

* *p*<0.05 *vs.* before.

### H_2_O_2_ induces augmentation of apneic responses to VLCF stimulants

**Figure 1:**
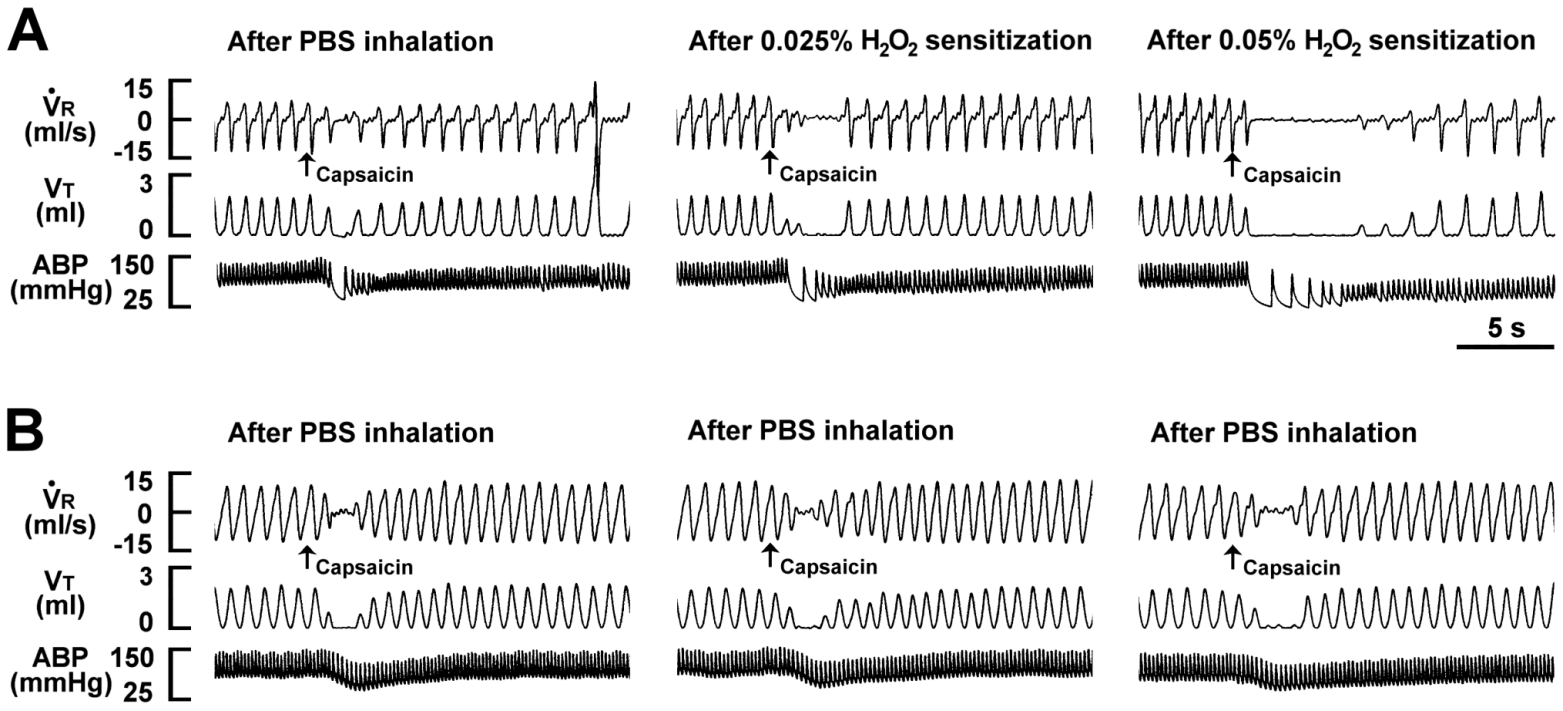
Ventilatory responses to intravenous capsaicin before and after inhalations of aerosolized H_2_O_2_ or PBS. Tracings in panels A and B were recorded from two separate rats. Aerosolized H_2_O_2_ (0.025 and 0.05%) or PBS was spontaneously inhaled into the lungs for 90 s. Three consecutive inhalations were performed and an elapse time of 90 min was allowed between any two inhalations. Capsaicin (0.5 µg/kg; a TRPV1 agonist) was injected into the jugular vein as a bolus at 1 min after inhalation of H_2_O_2_ or PBS had terminated as indicated by the arrows. VR, respiratory flow; VT, tidal volume; ABP, arterial blood pressure. Note that H_2_O_2_ augmented the apneic responses to capsaicin, while PBS had no such effect.

**Figure 2:**
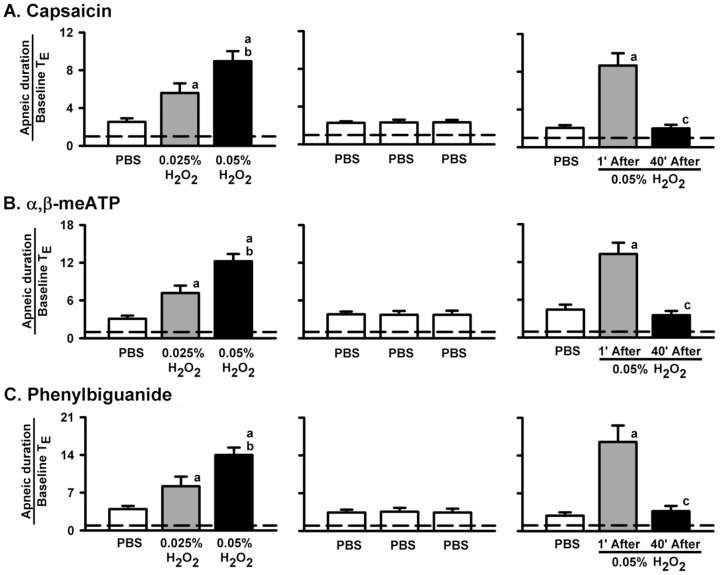
H_2_O_2_ induces augmentation of the apneic responses to intravenous capsaicin, α,β-meATP or phenylbiguanide. Data in each panel were obtained from one individual group of rats with three consecutive inhalations. Capsaicin (0.5 µg/kg; a TRPV1 receptor agonist), α,β-meATP (10 µg/kg; a P2X receptor agonist), and phenylbiguanide (5 µg/kg; a 5-HT_3_ receptor agonist) were separately injected into the jugular vein at 1 min (all panels) or 40 min (right panel) after termination of inhalation of H_2_O_2_ or PBS for 90 s. The longest expiratory duration (TE) occurring during the first 10 s after agonist injection was divided by the baseline TE to yield the apneic ratio. The horizontal dashed lines indicate an apneic ratio of 1 (no response). ^a^ *p*<0.05 *vs.* response before H_2_O_2_ sensitization; ^b^ *p*<0.05 *vs.* response after sensitization by 0.025% H_2_O_2_; ^c^ *p*<0.05 *vs.* response 1 min after sensitization by 0.05% H_2_O_2_. Data in each group are the mean ± SE of eight rats. Note that the H_2_O_2_ augmented the apneic responses to all agonists in a concentration-dependent manner, while PBS had no such effect. The sensitizing effect of H_2_O_2_ existed 1 min after, but disappeared 40 min after termination of inhalation of H_2_O_2_. See legend of figure 1 for detail.

We first investigated the ventilatory responses to VLCF stimulants before and after airway sensitization by H_2_O_2_. As shown in figure 1, one min following termination of the inhalation of PBS, the intravenous injection of capsaicin, a TRPV1 receptor agonist [21], resulted in the immediately (within 1 or 2 breaths) evoking of an apneic response, which was manifested by a prolongation of TE (*left* panels). This apneic response resulted in an increase in the apneic ratio (Figure 2A), an index reflecting the magnitude of the response. This apneic response was augmented by a subsequent inhalation of 0.025% (Figure 1A, *middle* panel) or 0.05% aerosolized H_2_O_2_ (Figure 1A, *right* panel) in the same animal. As a group, the apneic ratio after sensitization with 0.05% H_2_O_2_ was significantly greater than that after sensitization with 0.025% H_2_O_2_, which in turn was significantly greater than the control response (Figure 2A, *left* panel). In contrast, this apneic response was unaltered following three consecutive inhalations of PBS (Figures 1B and 2A, *middle* panel). Notably, the potentiating effect observed at 1 min following inhalation of 0.05% H_2_O_2_ had disappeared at 40 min following inhalation (Figure 2A, *right* panel). Similar results were obtained when α,β-meATP (Figure 2B), a P2X receptor agonist [22], and phenylbiguanide (Figure 2C), a 5-HT_3_ receptor agonist [17], were used individually as the VLCF stimulants.

### Role of VLCFs in the potentiating effect of H_2_O_2_ on apneic responses to VLCF stimulants

**Figure 3:**
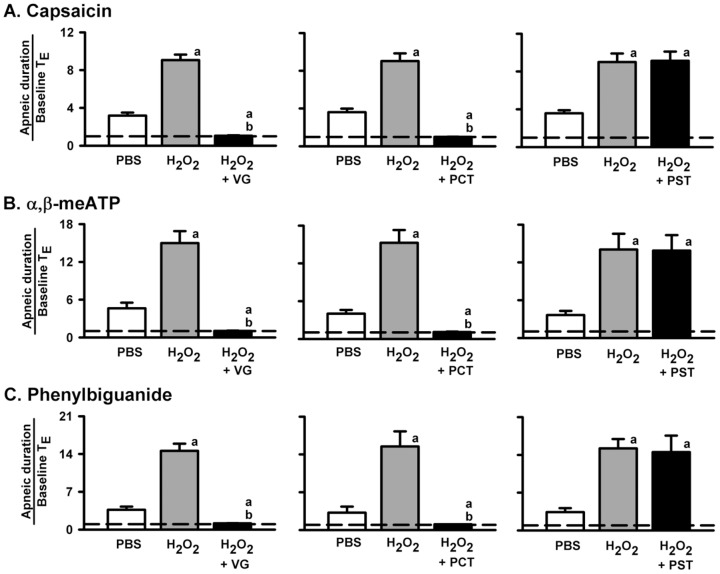
Role of vagal lung C-fibers in the sensitizing effect of H_2_O_2_ on the apneic responses to intravenous agonists. Data in each panel were obtained from one individual group of rats. Each rat received three consecutive inhalations. Bilateral cervical vagatomy (VG) is a treatment that blocks all vagal afferent pathways. Perivagal capsaicin treatment (PCT; 250 µg/ml) is known to block the neural conduction of vagal lung C-fibers. Perivagal sham treatment (PST) used the vehicle of capsaicin as a substitute for capsaicin. Intravenous agonist injection of capsaicin (A), α,β-meATP (B) or phenylbiguanide (C) was performed at 1 min after inhalation of H_2_O_2_ (0.05%) or PBS. Each vagal treatment was made 30 min prior to the second H_2_O_2_ sensitization. An elapsed time of 90 min was allowed between any two inhalations. ^a^ *p*<0.05 *vs.* response before H_2_O_2_ sensitization; ^b^ *p*<0.05 *vs.* response before vagal treatment. Data in each group are the mean ± SE of eight rats. Note that VG or PCT totally abolished the apneic responses to the agonists, while PST had no effect on either the apneic responses to the agonists or the sensitizing effect of H_2_O_2_. See legends of figures 1 and 2 for further explanations of H_2_O_2_ sensitization, function of agonist, and calculation of the apneic ratio.

We performed three different vagus nerve treatments to assess the role of VLCFs in airway sensitization by H_2_O_2_. As shown in figure 3, the apneic responses to capsaicin (Figure 3A), α,β-meATP (Figure 3B) or phenylbiguanide (Figure 3C) were totally abolished by either bilateral cervical vagatomy (*left* panels; a treatment that blocks all vagal afferent pathways) or by perivagal capsaicin treatment (*middle* panels; a treatment that blocks neural conduction of VLCFs) after airway sensitization with 0.05% H_2_O_2_. In contrast, the potentiating effect of 0.05% H_2_O_2_ on the apneic responses to these three VLCF stimulants was unaffected by perivagal sham treatment (*right* panels).

### Role of ROS in the augmentation of the apneic responses to VLCF stimulants

**Figure 4:**
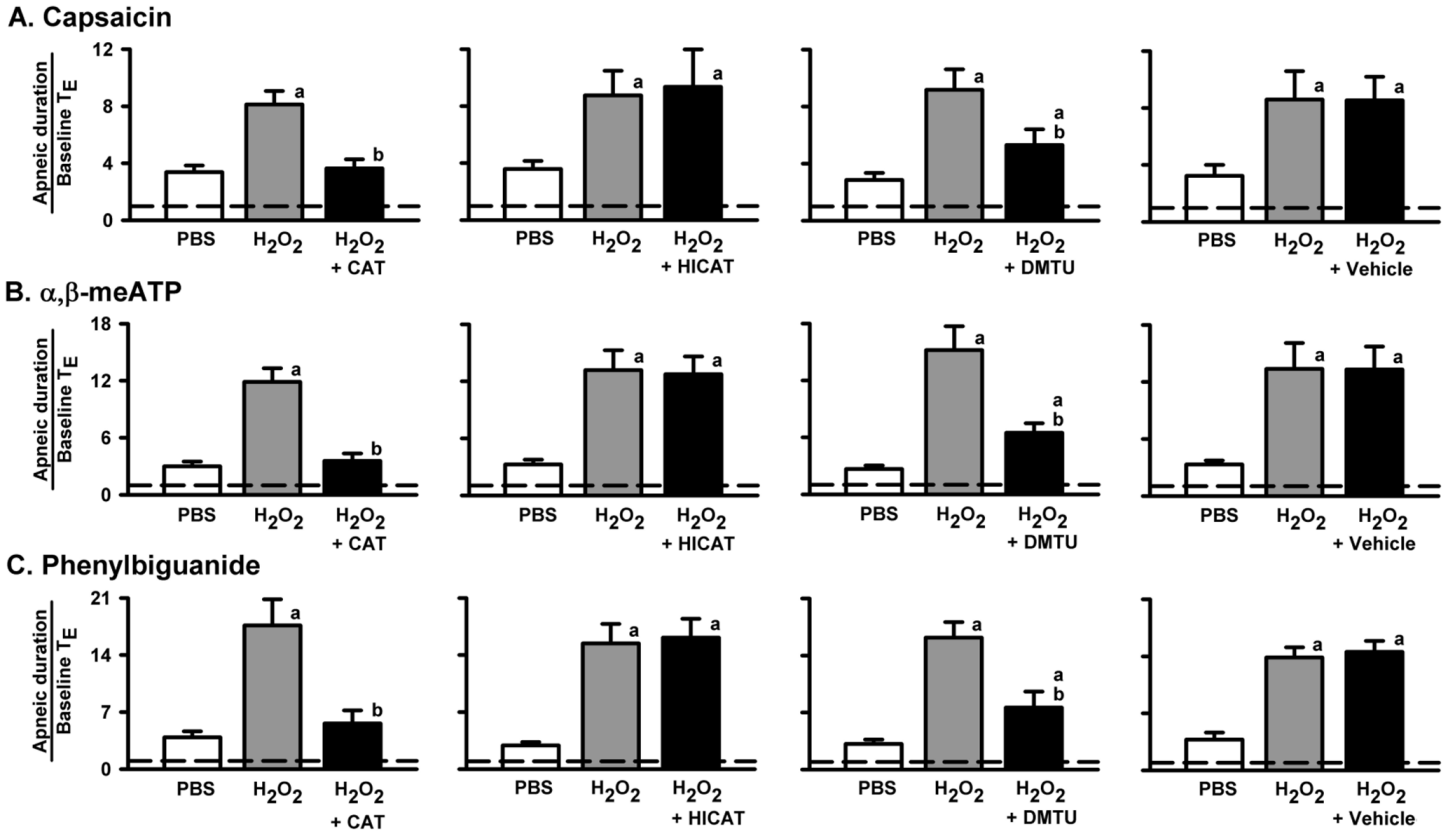
Role of ROS in the sensitizing effect of H_2_O_2_ on the apneic responses to intravenous agonists. Data in each panel were obtained from one individual group of rats. Each rat received three consecutive inhalations. Catalase (CAT) is an enzyme that is able to degrade H_2_O_2_, while heat-inactivated catalase (HICAT) serves as the control treatment. Dimethylthiourea (DMTU) is a ·OH scavenger, while its vehicle serves as the control treatment. Intravenous agonist injection of capsaicin (A), α,β-meATP (B) or phenylbiguanide (C) was performed at 1 min after inhalation of H_2_O_2_ (0.05%) or PBS. Pretreatment was made 10 min prior to the second H_2_O_2_ sensitization by delivery of aerosolized CAT (13500 IU/ml) or HICAT (13500 IU/ml) into lower airways for 5 min, or by slow infusion of DMTU (1 g/kg) or its vehicle into the jugular vein over a 10-min duration. An elapsed time of 90 min was allowed between any two inhalations. ^a^ *p*<0.05 *vs.* response before H_2_O_2_ sensitization; ^b^ *p*<0.05 *vs.* response before CAT or DMTU pretreatment. Data in each group are the mean ± SE of eight rats. Note that the sensitizing effect of H_2_O_2_ was totally abolished by CAT and was partly reduced by DMTU, while HICAT or vehicle of DMTU had no such effect. See legends of figures 1 and 2 for further explanations of H_2_O_2_ sensitization, function of agonist, and calculation of the apneic ratio.

We pretreated the animals with catalase (an enzyme to degrade H_2_O_2_), heat-inactivated catalase, dimethylthiourea (a •OH scavenger), or the vehicle of dimethylthiourea in order to investigate whether the potentiating effect was specific to the action of H_2_O_2_. As shown in figure 4, the potentiating effect of 0.05% H_2_O_2_ on the apneic responses to capsaicin (Figure 4A), α,β-meATP (Figure 4B) or phenylbiguanide (Figure 4C) was completely prevented by pretreatment with catalase (panels in *1^st^ column*) and was partially reduced by dimethylthiourea (panels in *3^rd^ column*), but was unaltered by pretreatment with heat-inactivated catalase (panels in *2^nd^ column*) or when the vehicle of dimethylthiourea was used (panels in *4^th^ column*).

### Role of the TRPV1, TRPA1, and P2X receptors in the potentiating effect of H_2_O_2_ on apneic responses to VLCF stimulants

**Figure 5:**
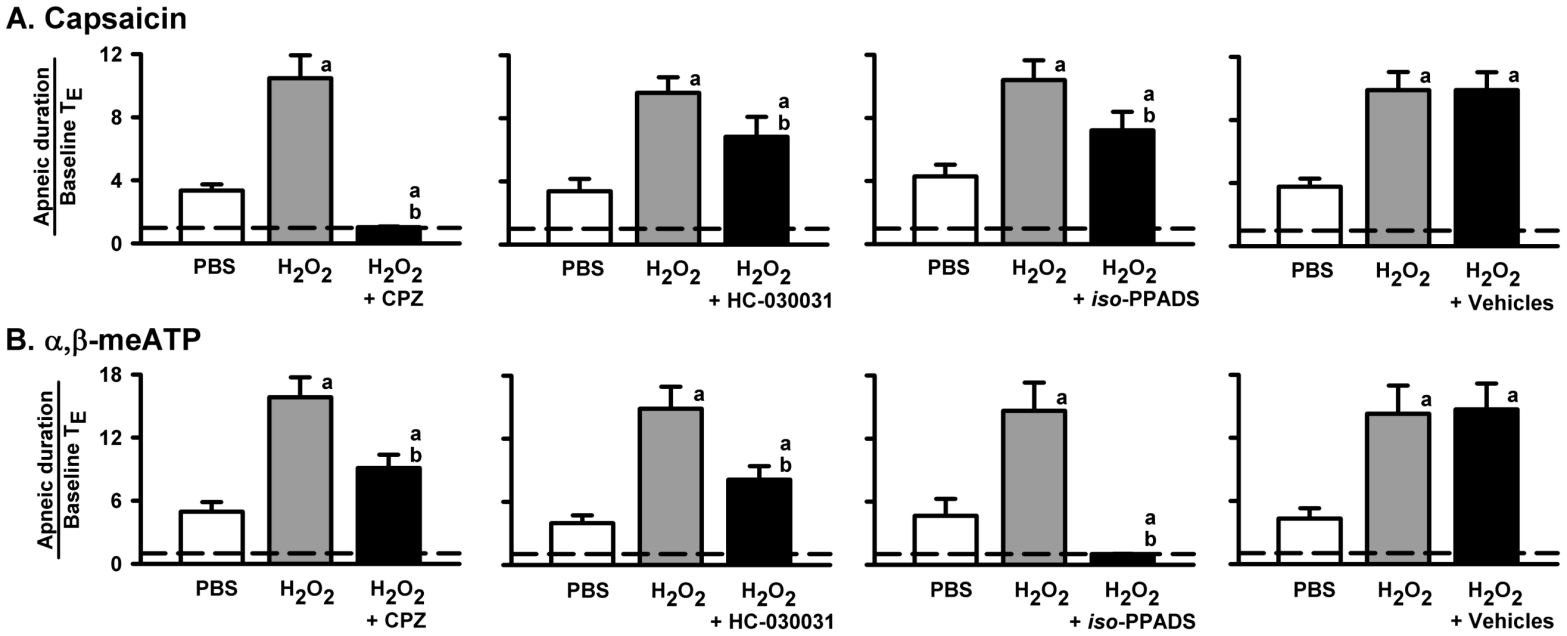
Role of TRPV1, TRPA1, and P2X receptors in the sensitizing effect of H_2_O_2_ on the apneic responses. Data in each panel were obtained from one individual group of rats. Each rat received three consecutive inhalations. Capsazepine (CPZ), HC-030031, and *iso*-PPADS are TRPV1, TRPA1, and P2X receptor antagonists, respectively. Intravenous agonist injection of capsaicin (A) and α,β-meATP (B) was performed at 1 min after inhalation of H_2_O_2_ (0.05%) or PBS. Pretreatments with CPZ (3 mg/kg), HC-030031 (3 mg/kg), *iso*-PPADS (15 mg/kg), and their vehicles were made by intravenous injection at 2, 10, 15, and 15 min, respectively, prior to the second H_2_O_2_ sensitization. An elapsed time of 90 min was allowed between any two inhalations. ^a^ *p*<0.05 *vs.* response before H_2_O_2_ sensitization; ^b^ *p*<0.05 *vs.* response before antagonist pretreatment. Data in each group are the mean ± SE of eight rats. Note that pretreatment with each antagonist partially attenuated the sensitizing effect of H_2_O_2_ on the apneic responses to other agonists, while pretreatment with vehicles had no such effect. Also, pretreatment with each antagonist totally abolished the apneic responses to corresponding agonists. See legends of figures 1 and 2 for further explanations of H_2_O_2_ sensitization, function of agonist, and calculation of the apneic ratio.

**Figure 6:**
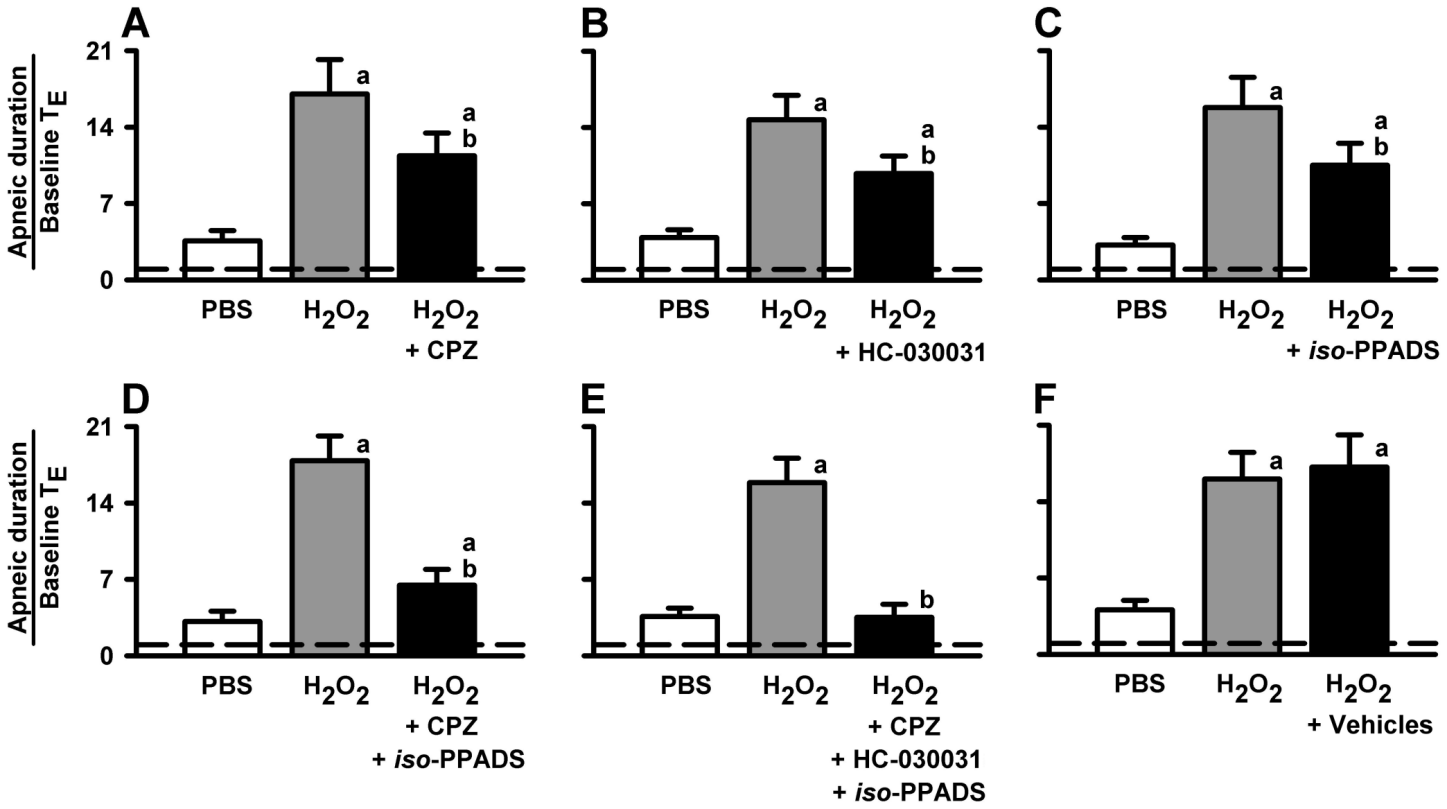
The sensitizing effect of H_2_O_2_ on the apneic responses is mediated through TRPV1, TRPA1, and P2X receptors. Data in each panel were obtained from one individual group of rats. Each rat received three consecutive inhalations. Capsazepine (CPZ), HC-030031, and *iso*-PPADS are TRPV1, TRPA1, and P2X receptor antagonists, respectively. Intravenous agonist injection of phenylbiguanide was performed at 1 min after inhalation of H_2_O_2_ (0.05%) or PBS. Pretreatments with CPZ (A), HC-030031 (B), *iso*-PPADS (C), a combination of CPZ and *iso*-PPAD (D; CPZ+*iso*-PPAD), a combination of three antagonists (E; CPZ+HC-030031+*iso*-PPAD), and their vehicles (F) were made by intravenous injection at 2, 10, 15, 15, 15, and 15 min, respectively, prior to the second H_2_O_2_ sensitization. An elapsed time of 90 min was allowed between any two inhalations. ^a^ *p*<0.05 *vs.* response before H_2_O_2_ sensitization; ^b^ *p*<0.05 *vs.* response before antagonist pretreatment. Data in each group are the mean ± SE of eight rats. Note that the sensitizing effect of H_2_O_2_ on the apneic responses to phenylbiguanide was partially attenuated by pretreatment with each antagonist, but was totally abolished by pretreatment with a combination of all three antagonists. Pretreatment with vehicles had no such effect. See legends of figures 1, 2, and 5 for further explanations of H_2_O_2_ sensitization, function of agonist or antagonists, and calculation of the apneic ratio.

We pretreated the animals with capsazepine (a TRPV1 receptor antagonist), HC-030031 (a TRPA1 receptor antagonist) or *iso*-PPADS (a P2X receptor antagonist) in order to investigate the role of these receptors in the airway sensitization by H_2_O_2_. As shown in figure 5, pretreatment with HC-030031 or *iso*-PPADS partially attenuated the potentiating effect of 0.05% H_2_O_2_ on the apneic responses to capsaicin, while pretreatment with capsazepine totally blocked the apneic response to capsaicin (*upper* panels). Similarly, pretreatment with capsazepine or HC-030031 partially attenuated the potentiating effect of 0.05% H_2_O_2_ on the apneic responses to α,β-meATP, while pretreatment with *iso*-PPADS completely prevented the apneic response to α,β-meATP (*lower* panels). Furthermore, pretreatment with the vehicles of these antagonists did not affect the potentiating effect of 0.05% H_2_O_2_ on the apneic responses to capsaicin or α,β-meATP. Moreover, as shown in figure 6, pretreatment with capsazepine (Figure 6A), HC-030031 (Figure 6B) or *iso*-PPADS (Figure 6C) partially attenuated the potentiating effect of 0.05% H_2_O_2_ on the apneic responses to phenylbiguanide. The suppression of the potentiating effect of 0.05% H_2_O_2_ by pretreatment with capsazepine alone (a reduction of 37.8%±10.5% of the control) or *iso*-PPADS alone (a reduction of 43.6%±8.6% of the control) was found to be further enhanced by pretreatment with a combination of these two antagonists (Figure 6D) (a reduction of 77.4%±4.5% of the control; *p*<0.05 *vs.* capsazepine alone or *iso*-PPADS alone). Notably, pretreatment with a combination of all three of these antagonists totally prevented the potentiating effect of 0.05% H_2_O_2_ on the apneic responses to phenylbiguanide (Figure 6E) (a reduction of 96.7%±6.2%), whereas pretreatment with their vehicles failed to produce such an effect (Figure 6F).

### Role of the TRPV1, TRPA1, and P2X receptors in the potentiating effect of H_2_O_2_ on the VLCF responses to stimulants

**Figure 7:**
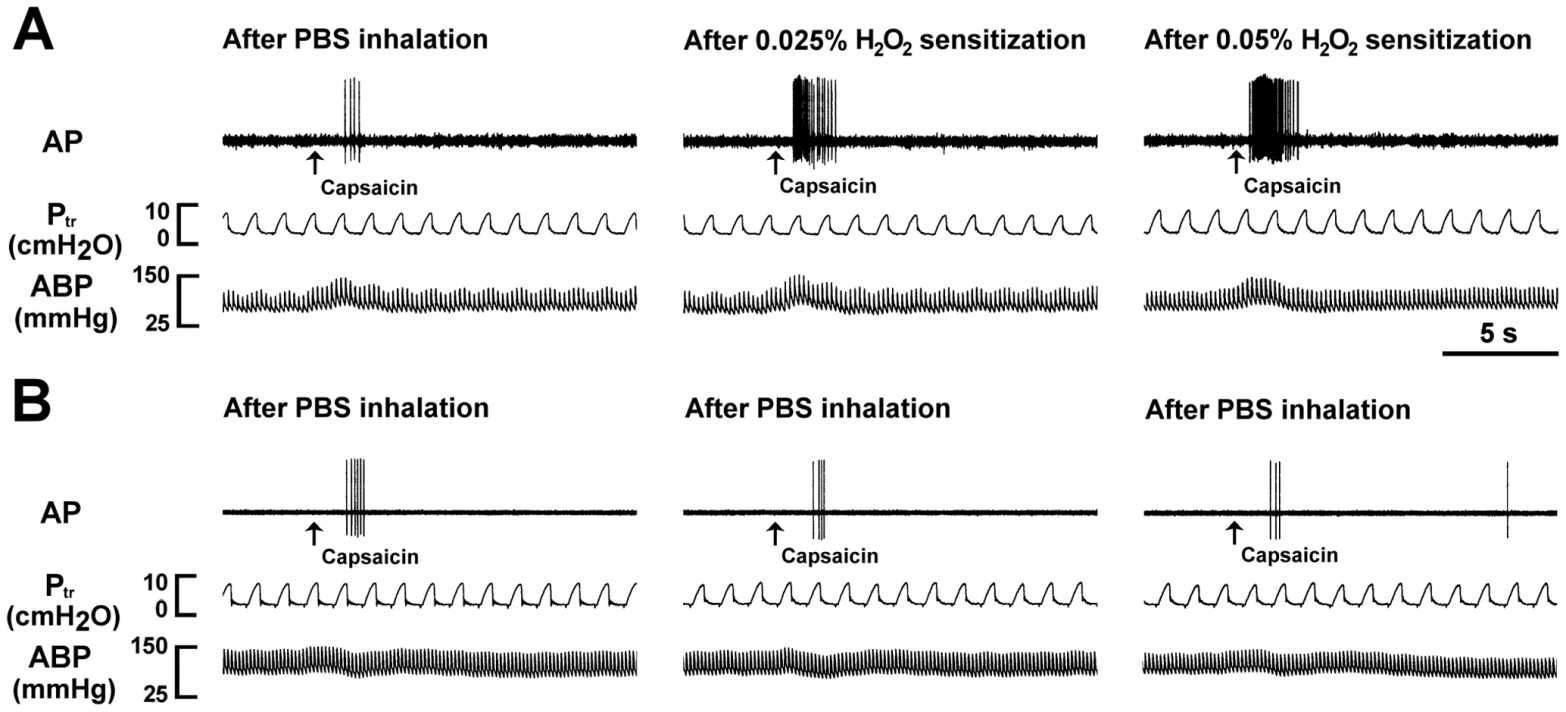
Responses of vagal lung C-fibers to intravenous capsaicin after inhalations of aerosolized H_2_O_2_ or PBS. Tracings in panels A and B were recorded from two separate rats. Aerosolized H_2_O_2_ (0.025% and 0.05%) or PBS was delivered into the lungs by the respirator for a period of 90 s. Capsaicin (0.5 µg/kg; a TRPV1 agonist) was injected into the jugular vein as a bolus 1 min after termination of inhalation of H_2_O_2_ or PBS as indicated by the arrows. Three consecutive inhalations were performed and an elapsed time of 90 min was allowed between any two inhalations. AP, action potential; P_tr_, tracheal pressure; ABP, arterial blood pressure. Note that H_2_O_2_ augmented the afferent responses to capsaicin, while PBS had no such effect.

**Figure 8:**
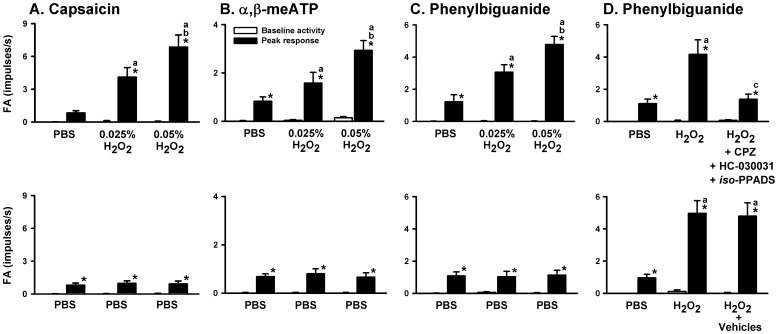
The sensitizing effect of H_2_O_2_ on vagal lung C-fibers is mediated through TRPV1, TRPA1, and P2X receptors. Data in each panel were obtained from one individual group of rats. Each rat received three consecutive inhalations. Panels A, B, and C show responses of vagal lung C-fibers to intravenous injection of capsaicin, α,β-meATP or phenylbiguanide, respectively, before and after inhalations of aerosolized H_2_O_2_ or PBS. Panel D shows responses of vagal lung C-fibers to intravenous injection of phenylbiguanide before and after inhalations of aerosolized H_2_O_2_ or PBS. Animals were pretreated with a combination of capsazepine (CPZ), HC-030031, and *iso*-PPADS, or their vehicles prior to the last inhalation. Baseline fiber activity (FA) was calculated as the average value over the 10-s period immediately preceding the agonist injection. The peak response of FA was defined as the maximal value averaged over 5 s immediately after the agonist injection. *p<0.05 *vs.* baseline activity; ^a^ *p*<0.05 *vs.* response before H_2_O_2_ sensitization; ^b^ *p*<0.05 *vs.* response after sensitization by 0.025% H_2_O_2_ (A–C); ^c^ *p*<0.05 *vs.* response before pretreatment with antagonists (D). Data are mean ± SE of 8 fibers from eight rats for each group. Note that H_2_O_2_ augmented the afferent responses to all agonists tested, while PBS had no such effect. Additionally, the sensitizing effect of H_2_O_2_ was totally prevented by pretreatment with a combination of all three antagonists, but was unaffected by pretreatment with their vehicles. See legends of figures 2, 5, and 7 for further explanations of H_2_O_2_ sensitization and function of agonist or antagonists.

We investigated the afferent responses of VLCFs to stimulants before and after airway sensitization by H_2_O_2_. As shown in figure 7, one min following termination of the inhalation of PBS, intravenous injection of capsaicin immediately (within 1 s or 2 s) evoked an intense burst of discharge (*left* panels). This afferent response to capsaicin was exaggerated by the subsequent inhalation of 0.025% (*middle* panel) or 0.05% aerosolized H_2_O_2_ (*right* panel) in the same animal. As a group, the afferent response to capsaicin after sensitization by 0.05% H_2_O_2_ was significantly greater than that after sensitization by 0.025% H_2_O_2_, which in turn was significantly greater than the control response (Figure 8A, *left upper* panel). In contrast, the afferent response to capsaicin was unaltered following three consecutive inhalations of PBS (Figures 7B and 8A, *left lower* panel). Similar results were obtained when α,β-meATP (Figure 8B) or phenylbiguanide (Figure 8C) was used as the VLCF stimulants. Notably, pretreatment with a combination of capsazepine, HC-030031, and *iso*-PPADS totally prevented the potentiating effect of 0.05% H_2_O_2_ on the afferent responses of VLCFs to phenylbiguanide (Figure 8D, *upper* panel), whereas pretreatment with vehicle failed to produce such effect (Figure 8D, *lower* panel). In contrast to the responses to chemical stimulation, inhalation of 0.05% H_2_O_2_ did not alter the VLCF responses to mechanical stimulation induced by lung inflation (tracheal pressure = 30 cmH_2_O) (Supporting Information Figure S1).

### Levels of ROS in the BALF after inhalation of PBS or H_2_O_2_

The level of ROS in BALF, when sampled at 60 s after termination of the inhalation of aerosolized 0.05% H_2_O_2_, was 91.6±4.7 units/s, which was significantly greater than that after termination of the inhalation of aerosolized PBS (79.2±2.6 units/s; *p*<0.05).

## Discussion

The results of this study demonstrate that inhalation of aerosolized 0.05% H_2_O_2_ for 90 s in anesthetized rats potentiated the apneic responses to intravenous injection of three chemical stimulants: capsaicin, α,β-meATP, and phenylbiguanide. The potentiating effect of H_2_O_2_ on the apneic responses to these stimulants was reversible because it disappeared at 40 min after termination of inhalation. It is also specific to the action of ROS because it was totally abolished by catalase and is partially prevented by dimethylthiourea. The difference between the suppressive effects of catalase and dimethylthiourea may be due to the fact that catalase removes H_2_O_2_, whereas dimethylthiourea only scavenges the derived product •OH. The apneic responses to these three stimulants were also abolished by vagatomy or perivagal capsaicin treatment, which suggests the responses are reflex consequences mediated through the VLCFs. Indeed, our electrophysiological studies have revealed that airway delivery of aerosolized 0.05% H_2_O_2_ for 90 s also potentiated the VLCF responses to these three stimulants. The potentiating effect on the apneic responses to capsaicin was found to be partially attenuated by HC-030031 and *iso*-PPADS independently, which suggests the involvement of both the TRPA1 and P2X receptors. The potentiating effect on the apneic responses to α,β-meATP was also partially reduced by capsazepine and HC-030031 independently, indicating the involvement of both the TRPV1 and TRPA1 receptors. The involvement of all three types of receptors was further confirmed by the findings that the potentiating effect on both reflex and VLCF responses to phenylbiguanide was totally abolished when all three antagonists were used in combination. Certainly, this does mean that that all 3 antagonists were needed to abolish the potentiating effect. The effectiveness of capsazepine, HC-030031, and *iso*-PPADS as receptor antagonists was also demonstrated. Finally, our chemiluminescence analysis indicated that inhalation of 0.05% H_2_O_2_ did indeed increase the lung level of ROS. Collectively, these results suggest that an increase in lung ROS may sensitize the VLCFs and this then leads to exaggerated reflex responses in rats; in addition, it would seem that all three receptors, TRPV1, TRPA1, and P2X, play a central part in the development of this airway hypersensitivity.

It has been previously shown that acute intermittent hypoxia [14] and laryngeal insult with acid and pepsin [15], [16] are able to potentiate the afferent and/or reflex responses of the airway C-fibers to stimulants in rats; the potentiating effect of the former and latter insults were found to be mediated through the TRPA1 and P2X receptors, respectively. In these studies [14]–[16], these exaggerated responses could be prevented by pretreatment with antioxidants, indicating that there seemed to be an involvement of ROS in the sensitization of airway C-fibers and the development of airway hypersensitivity. However, questions remained as to whether the suppressive effects of the antioxidants in these studies resulted from a prevention of sensitization of the afferent fibers by the increased ROS or occurred because there was a removal of the basal function of ROS, which led to diminished afferent fiber sensitivity to these airway insults. Thus, our findings provide the first direct evidence in support of the notion that increased lung ROS may sensitize VLCFs and that this then leads to the airway hypersensitivity. Our findings are consistent with observations that intravesical administration of H_2_O_2_ is able to induce detrusor overactivity via the C-fiber pathway and that this is also prevented by catalase and dimethylthiourea [37]. It is known that asthmatic lungs exhibit excess production of ROS, which have been suggested to play a role in the pathogenesis of asthma [4], [5]. Our findings that increased lung ROS may induce exaggerated reflex responses to stimulants due to sensitization of the VLCFs thus provide a mechanism to explain the important role of ROS.

The mechanism by which inhalation of 0.05% H_2_O_2_ dramatically enhances the sensitivity of VLCFs to different chemical stimulants, which, in turn, leads to exaggerated reflex responses, remains unclear. In this study, immediately after inhalation of 0.05% H_2_O_2_ for 90 s, a slight increase in baseline C-fiber activity could be noted, which supports the presence of a mild stimulation of the VLCFs. It should be noted that this increase is small in comparison to the intense stimulation of VLCFs that is evoked by inhalation of 0.4% H_2_O_2_ for 30 s, which also involves the TRPV1, TRPA1, and P2X receptors as reported in a number of recent studies [20], [21]. While several studies have reported that ROS may directly activate TRPA1 receptors, other investigations suggest that TRPV1 and P2X receptors may be activated downstream of other ROS-induced biological processes such as cellular destruction, lipid metabolism, and ATP release [24]. Perhaps, a mild and more persistent activation of these receptors by H_2_O_2_ is able to sensitize VLCFs. This notion is in agreement with previous findings indicating that a mild and prolonged activation of TRPV1 [28] and adenosine A1 receptors [38] by their agonists is able to lead to an augmentation of VLCF responses to chemical stimulants. This notion also gains supports from previous observations where it was demonstrated that the activation of bradykinin [39], prostanoid [40], histamine [41], and TRPA1 receptors [42] by their agonists is able to nonspecifically increase the excitability of VLCFs. In this context, our findings, which show that all three receptors, TRPV1, TRPA1, and P2X, take part in the development of this afferent sensitization, suggest that the process is Ca^2+^-dependent; this is because these receptors are non-selective cation channels that are mainly permeable to Ca^2+^ [10], [24]. Since capsaicin, α,β-meATP, and phenylbiguanide are TRPV1, P2X, 5-HT_3_ receptor agonists, respectively [17], [21], [22], it appears that increased lung ROS produces a nonspecific increase in the electrical excitability of the VLCFs. Alternatively, activation of these receptors by H_2_O_2_ may possibly promote a common cellular mechanism that sensitizes the functioning of these ionotropic receptors. Indeed, exposure to oxidizing agents [43] or H_2_O_2_ [44] has been shown to potentiate the responses of transfected TRPV1 receptors in HEK293 cells to capsaicin and heat, independently. Excessive activation of TRPV1 and TRPA1 receptors has been suggested as an obligatory step in the development of increased pain hypersensitivity *in vivo* [37], [44]–[46]. P2X receptors have also been shown to mediate afferent hypersensitivity in the intestine [47], in the heart [48], and in skin [49] under inflammatory conditions.

Stimulation and sensitization are two different pathophysiological features of VLCFs. The stimulation of VLCFs by stimuli evokes reflex responses, whereas the sensitization of VLCFs by sensitizing agents leads to augmented reflex responses to stimuli [1], [2], [9], [10]. Our previous studies [20]–[23] focused on the mechanism underlying the stimulation of VLCFs by a relatively high concentration of H_2_O_2_ concentration, whereas this study focused on the mechanism underlying the sensitization of these afferents by a low concentration of H_2_O_2_. Thus, this study shows that, in additional to its stimulatory effect, H_2_O_2_ can be a sensitizing agent for VLCFs and this finding may advance of knowledge in the area of C-fiber-mediated airway hypersensitivity.

In asthmatic patients with inhaled corticosteroid therapy, the H_2_O_2_ concentration in exhaled breath condensate can be as high as 2 µM, which is 6.18×10^−6^% [7], [8]. This concentration is lower than that used in this study apparently. However, the H_2_O_2_ concentrations in both exhaled breath condensate and inhaled air do not allow us to know for certain what is the local concentration in the airways. The other difference is that asthmatic airways are constantly exposed to this increased concentration of H_2_O_2_, whereas we only observed the effect of inhalation of aerosolized H_2_O_2_ for 90 s.

In conclusion, the results from the present study clearly have demonstrated that there is a sensitizing effect of ROS on VLCFs in anaesthetized rats. This sensitizing effect results in a nonspecific increase in the electrical excitability of the VLCFs and augments the reflex apneic responses to chemical stimulants of VLCFs. This sensitizing effect appears to be mediated through activation of the TRPV1, TRPA1, and P2X receptors, which are presumably located at terminals of the VLCFs. Our findings thus provide support for the use of antioxidants as the therapy when treating asthmatic patients.

## Supporting Information

Figure S1 **Responses of vagal lung C-fibers to lung inflation after inhalations of aerosolized H_2_O_2_ or PBS.**
(DOC)

Table S1 **Average apneic responses to intravenous agonists before and after treatment with antioxidants or antagonists in 8 study groups.**
(DOC)

Table S2 **Pharmacological agents used in this study.**
(DOC)
