# Supplementary material for: Sensitization by Pulmonary Reactive Oxygen Species of Rat Vagal Lung C-Fibers: The Roles of the TRPV1, TRPA1, and P2X Receptors
Source: PLoS One. 2014 Apr 3;9(4):e91763. doi: 10.1371/journal.pone.0091763 (PMC3974698; doi:10.1371/journal.pone.0091763)
Supplement: Figure S1 — Responses of vagal lung C-fibers to lung inflation after inhalations of aerosolized H2O2 or PBS. (DOC) [file pone.0091763.s001.doc]

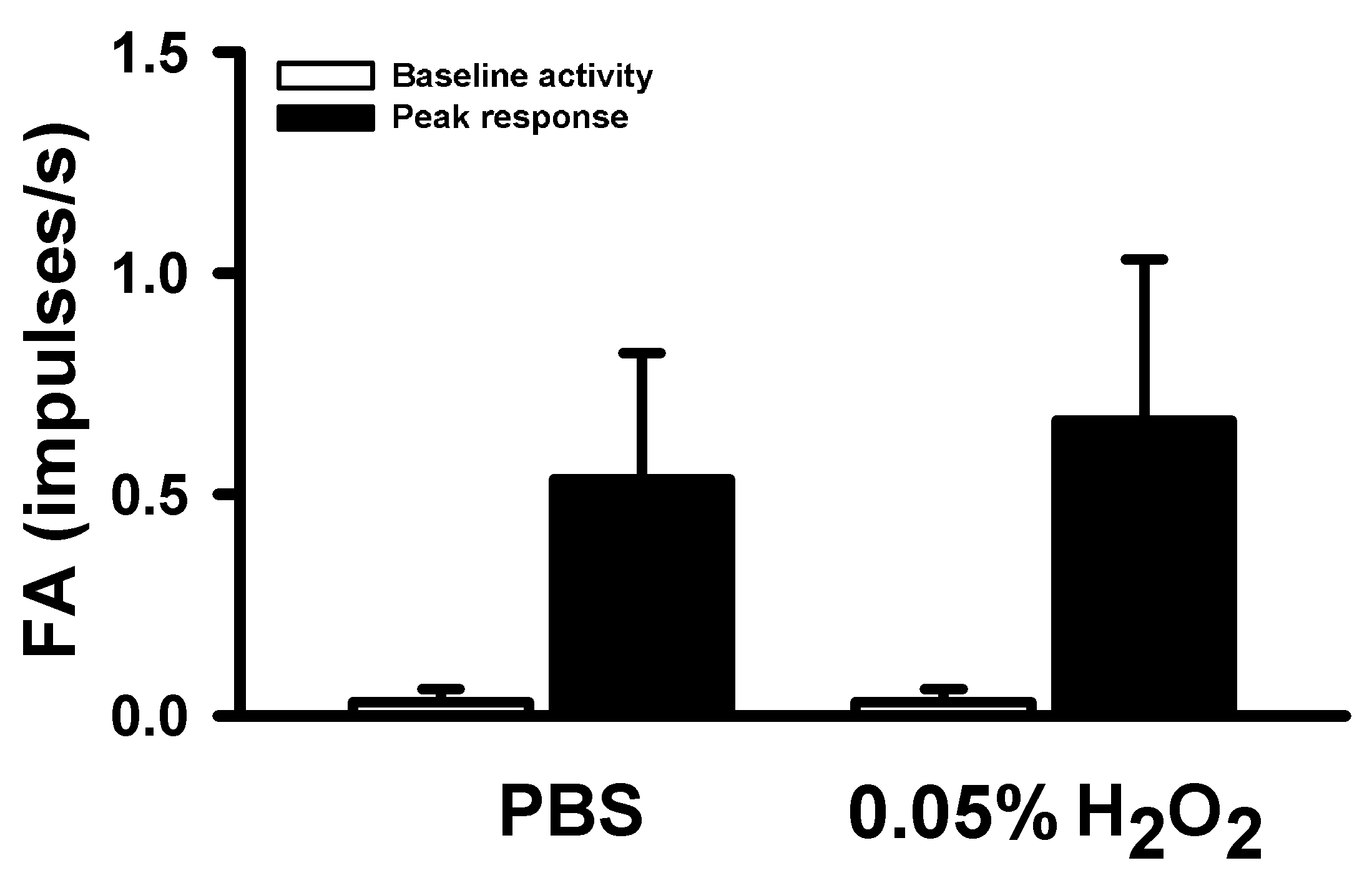


**Figure S1. Responses of vagal lung C-fibers to lung inflation after inhalations of aerosolized H_2_O_2_ or PBS.** Aerosolized H_2_O_2_ (0.05%) or PBS was delivered into the lungs by the respirator for a period of 90 s. Lung inflation was performed at 1 min after termination of inhalation of H_2_O_2_ or PBS. The lung was inflated with a constant air-flow (~ 12 ml/s) until the tracheal pressure reached 30 cmH_2_O; the lungs were then maintained at that pressure for 10 s after turning off the respirator. Baseline fiber activity (FA) was calculated as the average value over the 10-s period immediately preceding lung inflation. The peak response of FA was defined as the maximal value averaged over 5 s immediately after lung inflation. Data are mean ± SE of 8 fibers from eight rats. Note that afferent responses to lung inflation after inhalation of H_2_O_2_ did not statistically differ from those after inhalation of PBS.
