# Supplementary material for: Sensitization by Pulmonary Reactive Oxygen Species of Rat Vagal Lung C-Fibers: The Roles of the TRPV1, TRPA1, and P2X Receptors
Source: PLoS One. 2014 Apr 3;9(4):e91763. doi: 10.1371/journal.pone.0091763 (PMC3974698; doi:10.1371/journal.pone.0091763)
Supplement: Table S1 — Average apneic responses to intravenous agonists before and after treatment with antioxidants or antagonists in 8 study groups. (DOC) [file pone.0091763.s002.doc]

**Table S1.** **Average apneic responses to intravenous agonists before and after treatment with antioxidants or antagonists in 8 study groups.**

| Agonist | Antioxidant or Antagonist | Responses to Agonist (Apneic Ratio) | |
| --- | --- | --- | --- |
|  |  | Before Treatment | After Treatment |
| Capsaicin | CAT | 7.79 ± 1.12 | 7.64 ± 1.25 |
| Capsaicin | DMTU | 7.18 ± 0.59 | 6.72 ± 0.60 |
| Capsaicin | CPZ | 8.84 ± 1.25 | 1.05 ± 0.05* |
| Polygodial | HC-030031 | 10.86 ± 1.62 | 1.11 ± 0.08* |
| α,β-methylene-ATP | *iso*-PPADS | 10.62 ±1.28 | 1.13 ± 0.11* |
| Phenylbiguanide | Tropisetron | 15.05 ± 2.01 | 0.98 ± 0.02* |
| Phenylbiguanide | CPZ+*iso*-PPADS+HC-030031 | 13.63 ± 3.10 | 15.11 ± 4.54 |
| Phenylbiguanide | Vehicles | 12.54 ± 2.05 | 12.41 ± 1.39 |

Capsaicin (1 μg/kg; a TRPV1 receptor agonist); polygodial (100 μg/kg; a TRPA1 receptor agonist); α,β-methylene-ATP (15 μg/kg; a P2X receptor agonist); phenylbiguanide (6 μg/kg; a 5-HT_3_ receptor agonist); CAT, catalase (13500 IU/ml; an enzyme that degrades H_2_O_2_); DMTU, dimethylthiourea (1 g/kg; a ∙OH scavenger); CPZ, capsazepine (3 mg/kg; a TRPV1 receptor antagonist); HC-030031 (3 mg/kg; a TRPA1 receptor antagonist); tropisetron (15 μg/kg; a 5-HT_3_ receptor antagonist); *iso*-PPADS, *iso*-pyridoxalphosphate-6-azophenyl-2',5'- disulphonate (15 mg/kg; a P2X receptor antagonist). CPZ+*iso*-PPADS+HC-030031 represent a combination of CPZ, *iso*-PPADS, and HC-030031. Vehicles represent a combination of vehicles of CPZ, *iso*-PPADS, and HC-030031. The longest expiratory duration (TE) occurring during the first 10 breaths after agonist injection or lung inflation was divided by the baseline TE to yield the apneic ratio. * *p*<0.05 *vs*. the responses before intervention, *p*< 0.05. Data in each group are the means ± SE from 8 rats. Note that CPZ, HC-030031, and *iso*-PPADS totally blocked the apneic response to their corresponding agonists, suggesting the effectiveness of these antagonists. Also, CPZ+*iso*-PPADS+HC-030031 did not affect the apneic response to phenylbiguanide, suggesting the selectivity of these antagonists. Furthermore, CAT and DMTU did not affect the apneic response to capsaicin, suggesting that these antioxidants did not produce possible toxic effects on vagal lung C-fibers.
