# Supplementary material for: Sensitization by Pulmonary Reactive Oxygen Species of Rat Vagal Lung C-Fibers: The Roles of the TRPV1, TRPA1, and P2X Receptors
Source: PLoS One. 2014 Apr 3;9(4):e91763. doi: 10.1371/journal.pone.0091763 (PMC3974698; doi:10.1371/journal.pone.0091763)
Supplement: Table S2 — Pharmacological agents used in this study. (DOC) [file pone.0091763.s003.doc]

**Table S2. Pharmacological agents used in this study**.

| Drug Name | Drug function | Dose/Route | Vehicle of drug | Reference |
| --- | --- | --- | --- | --- |
| Capsaicin | TRPV1 receptor  agonist | 0.5 μg/kg; i.v. | Tween 80 (0.12%),  ethanol (0.12%), saline | 21, 22, 28 |
| α,β-methylene-ATP | P2X receptor  agonist | 10 μg/kg; i.v. | saline | 18, 22 |
| Phenylbiguanide | 5-HT_3_ receptor agonist | 5 μg/kg; i.v. | saline | 17, 19, 22 |
| Catalase | H_2_O_2_ scavenger | 13500 IU/ml;  aerosol inhalation | distilled water,  thymol (0.05%), PBS | 21, 23 |
| Dimethylthiourea | ∙OH scavenger | 1 g/kg; i.v. | saline | 14, 21, 23, 36 |
| Capsazepine | TRPV1 receptor  antagonist | 3 mg/kg; i.v. | dimethyl sulfoxide (7.6%),  Tween 80 (9.2%),  ethanol (9.2%), saline | 20, 21, 22 |
| *iso*-pyridoxalphosphate-6-azophenyl-2',5'-  disulphonate | P2X receptor  antagonist | 15 mg/kg; i.v. | saline | 18, 20, 22 |
| HC-030031 | TRPA1 receptor  antagonist | 3 mg/kg; i.v. | dimethyl sulfoxide (6.7%),  Tween 80 (18.3%),  ethanol (8.3%), saline | 14, 18, 20 |
